# The Impact of Clostridium Difficile Infections on In-Hospital Outcomes of Venous Thromboembolism (Deep Vein Thrombosis or Pulmonary Embolism) Hospitalizations

**DOI:** 10.7759/cureus.9195

**Published:** 2020-07-15

**Authors:** Khushali Jhaveri, Aniruddh Som, Sandeep A Padala, Salim Surani

**Affiliations:** 1 Internal Medicine, The Georgetown University Hospital/MedStar Washington Hospital Center, Washington, DC, USA; 2 Nephrology, Medical College of Georgia - Augusta University, Augusta, USA; 3 Internal Medicine, Corpus Christi Medical Center, Corpus Christi, USA; 4 Internal Medicine, University of North Texas, Dallas, USA

**Keywords:** clostridium difficle infection, pulmonary embolism, deep venous thrombosis, hospital admission, hospital length of stay

## Abstract

Background

Clostridium difficile infection (CDI) is associated with high mortality. Studies have shown an increased rate of venous thromboembolism (VTE) in patients with CDI. However, literature regarding the impact of CDI on outcomes of VTE-related hospitalizations is scarce. Our study aimed to assess the impact of CDI on in-hospital outcomes among VTE hospitalizations.

Methods

The 2016 National Inpatient Sample (NIS) was used to identify all adult hospitalizations in the United States with a primary discharge diagnosis of acute VTE. Hospitalizations with deep vein thrombosis (DVT) or pulmonary embolism (PE) were included under VTE. The sample was stratified based on the presence or absence of active CDI. Chi-square test and weighted Student’s t-test were used to analyze categorical and continuous variables, respectively. The adjusted odds ratio (OR) for clinical outcomes were calculated using multivariate logistic regression analysis. Subgroup analyses for DVT and PE hospitalizations were performed. All analyses were completed in SAS (SAS Institute Inc., Cary, NC), and a p-value of <0.05 was considered statistically significant.

Results

We identified 382,585 weighted hospitalizations for VTE. Among them, 0.8% had concomitant CDI. The presence of CDI was associated with a statistically significant increase in in-hospital mortality (6% vs. 3%), hospitalization cost ($147,356.5 vs. $55,193), and length of stay (13.7 vs. 5.4 days). There were more incidents of bleeding and acute respiratory failure requiring prolonged ventilation in patients with CDI. The odds of stroke were significantly higher in patients with CDI and DVT.

Conclusion

CDI independently increased in-hospital mortality in VTE. Preventing CDI in the VTE population may mitigate complications, improve in-hospital outcomes, and reduce treatment costs.

## Introduction

Clostridium difficile infection (CDI) is a leading cause of nosocomial infection, resulting in a significant healthcare burden, morbidity, and mortality [1]. According to the literature, there was a two-fold increase in the incidence of CDI among hospitalized adults in the United States between 2001 and 2010, with more recent data suggesting approximately 365,000 CDI cases reported in the United States annually [2].

It is well-known that the pathogenesis of venous thromboembolism (VTE) involves the Virchow’s triad: stasis, endothelial injury, and hypercoagulability. Various studies have shown an increased rate of VTE in patients with CDI, likely secondary to the formation of a proinflammatory state [3,4]. However, data is scarce regarding the impact of CDI on outcomes of VTE hospitalizations. It is possible that CDI leads to poorer outcomes in these patients, whether due to a direct effect of CDI (prolonged hospital stay and associated complications), or due to the proposed effect of CDI on coagulability, which might be seen in terms of sequelae of clot extension, anticoagulation failure, embolic events, or bleeding complications. In this study, we sought to assess the impact of the presence or absence of CDI on outcomes of VTE hospitalizations.

## Materials and methods

Data were extracted from the 2016 Healthcare Cost and Utilization Project’s (HCUP) National Inpatient Sample (NIS). The NIS is the largest publicly available database in the United States and approximates a 20% stratified sample of discharges from US hospitals. In our analysis, all patients of ≥18 years of age who were identified with VTE-related hospitalizations [pulmonary embolism (PE) or deep vein thrombosis (DVT)] were included. Furthermore, VTE hospitalizations with and without CDI were identified, and their clinically relevant outcomes were compared. Given the publicly available nature of the dataset, the study was exempted from institutional review board (IRB) approval.

All statistical analyses followed the sample design elements (clusters, strata, and weights) provided by the NIS [5]. Continuous variables were reported as weighted means ±standard error (SE), and categorical variables were reported as weighted numbers and percentages. The standard errors of weighted means were estimated by using the Taylor linearization method that incorporates the sample design. Length of stay and total cost of hospitalization were normalized by log-transformation for all analyses, and antilog-transformed results from the multivariate linear regression models were then reported. The total costs of hospitalization were inflation-adjusted for 2018 using Consumer Price Index data provided by the US Department of Labor.

The differences in outcomes variables between hospitalizations with and without CDI were compared using weighted Student’s t-tests for continuous variables, and Rao-Scott modified chi-square tests for categorical variables. A multivariate logistic regression model was used to estimate the odds ratio (OR) of clinical outcomes after adjusting for patient demographics, hospital bed size, hospital location/teaching status, insurance type, household income, and relevant comorbidities, and incorporating hospital as a random effect. Subgroup analyses were conducted separately for hospitalizations with PE and DVT, again comparing in-hospital outcomes for those with and without CDI. Unadjusted and adjusted ORs and their corresponding 95% confidence intervals (CI) were reported. All statistical analyses were performed using the SAS Survey Procedures (SAS 9.4; SAS Institute Inc., Cary, NC). Statistical significance was defined by two-sided p-values of <0.05.

## Results

We identified 382,585 admissions with VTE. Of those, 3,080 (0.8%) had a concomitant diagnosis of CDI. The VTE population with CDI had a higher mean age compared to the VTE population without CDI (66.2 vs. 63.1 years, respectively). No statistically significant difference was found in terms of gender, race, or mean household income between both populations (Table 1).

**Table 1:** Baseline characteristics of the study population VTE: venous thromboembolism; SE: standard error

Characteristics	VTE hospitalizations with Clostridium difficile infections		VTE hospitalizations without Clostridium difficile infections		P-value
N (unweighted)	616		76,517		
N (weighted)	3,080		382,585		
	Mean	SE	Mean	SE	
Age, years	66.2	0.6	63.1	0.1	
Length of stay, days	13.7	0.6	5.4	0.03	
Total cost of hospitalization, $	147,357	12,734	55,193	764	
	Weighted N	%	Weighted N	%	
Gender					
Male	1,405	45.60%	182,305	47.70%	0.32
Female	1,675	54.40%	200,280	52.30%	
Race/ethnicity					
White	2,250	73.10%	271,240	70.90%	0.2
Black	440	14.30%	69,680	18.20%	
Hispanic	235	7.60%	26,865	7%	
Asian or Pacific Islander	45	1.50%	4,075	1.10%	
Native American	15	0.50%	1,490	0.40%	
Other	95	3.10%	9,235	2.40%	
Insurance type					
Medicare	2,010	65.30%	202,675	53%	
Medicaid	365	11.90%	48,995	12.80%	
Private	570	18.50%	103,955	27.20%	
Self-pay	65	2.10%	14,240	3.70%	
Other	65	2.10%	11,170	2.90%	
Hospital region					
Northeast	645	20.90%	76,725	20.10%	0.001
Midwest	615	20%	85,945	22.50%	
South	1,135	36.90%	157,225	41.10%	
West	685	22.20%	62,690	16.40%	
Hospital location/teaching status					
Rural	125	4.10%	33,505	8.80%	
Urban non-teaching	850	27.60%	104,790	27.40%	
Urban teaching	2,105	68.30%	244,290	63.90%	
Hospital bed size					
Small	440	14.30%	71,310	18.60%	0.026
Medium	920	29.90%	110,225	28.80%	
Large	1,720	55.80%	201,050	52.60%	
Household income					
Q1	800	26%	116,220	30.40%	0.075
Q2	895	29.10%	97,850	25.60%	
Q3	750	24.40%	91,365	23.90%	
Q4	635	20.60%	77,150	20.20%	
In-hospital mortality	185	6%	11,385	3%	0.002
Comorbidities					
Renal failure	635	20.60%	50,745	13.30%	
Deficiency anemia	1,105	35.90%	82,910	21.70%	
Congestive heart failure	665	21.60%	47,070	12.30%	
Metastatic cancer	410	13.30%	28,715	7.50%	
Sepsis	250	8.10%	5,070	1.30%	
Paralysis	220	7.10%	12,380	3.20%	0.0002
Diabetes, complicated	415	13.50%	37,255	9.70%	0.005
Liver disease	185	6%	13,865	3.60%	0.021
Coagulopathy	370	12%	29,710	7.80%	0.001
Prior VTE	330	10.70%	51,570	13.50%	0.03
Hyperlipidemia	755	24.50%	112,925	29.50%	0.004
Obesity	400	13%	81,050	21.20%	
Smoking	325	10.60%	54,250	14.20%	0.004
Hypertension	1,820	59.10%	231,665	60.60%	0.47
Solid tumor without metastasis	240	7.80%	21,725	5.70%	0.058
Lymphoma	75	2.40%	4,960	1.30%	0.069
Rheumatoid arthritis/collagen vascular disease	140	4.50%	14,835	3.90%	0.422
Peripheral vascular disease	205	6.70%	19,965	5.20%	0.163
Chronic pulmonary disease	675	21.90%	89,545	23.40%	0.369
Diabetes, uncomplicated	390	12.70%	56,045	14.60%	0.131
Alcohol abuse	150	4.90%	13,820	3.60%	0.144

The VTE population with concomitant CDI was found to have higher prevalence of comorbidities compared to those without CDI, including renal failure (20.6% vs. 13.3%), congestive heart failure (35.9% vs. 21.7%), complicated diabetes (13.5% vs. 9.7%), metastatic cancer (13.3% vs. 7.5%), liver disease (6% vs. 3.6%), deficiency anemia (35.9% vs. 21.7%), coagulopathy (12% vs. 7.8%) and paralysis (7.1% vs. 3.2%). They were also more likely to present with sepsis (8.1% vs. 1.3%). However, the VTE population without CDI had a higher prevalence of hyperlipidemia (29.5% vs. 24.5%), obesity (21.2% vs. 13%), smoking (14.2% vs. 10.6%), and prior VTE (13.5% vs. 10.7%). VTE hospitalizations with CDI were found to have increased in-hospital mortality (6% vs. 3%, adjusted OR: 1.54, 95% CI: 1.09-2.17, p = 0.014), longer mean length of hospital stay (13.7 days vs. 5.4 days, adjusted OR: 2.10, 95% CI: 1.98-2.24, p: <0.001) and hospitalization cost ($147,356.5 vs. $55,193, adjusted OR: 2.01, 95% CI: 1.87-2.17, p: <0.001) (Table 2).

**Table 2:** In-hospital outcomes in VTE hospitalizations with and without Clostridium difficile infections *Adjusted for age, race, sex, insurance status, hospital characteristics, and all significant comorbidities listed in Table 1 †Parameter estimates represent the antilog of the b regression coefficients obtained from the log-transformed regression models VTE: venous thromboembolism; OR: odds ratio; CI: confidence interval; AKI: acute kidney injury; AKI-D: acute kidney injury requiring dialysis; DIC: disseminated intravascular coagulation; SE: standard error

Variables	VTE patients		
	No Clostridium difficile infections	Clostridium difficile infections	P-value
In-hospital mortality			
Incidence, %	3	6	
Adjusted OR (95% CI)*	Ref	1.54 (1.09–2.17)	0.014
AKI			
Incidence, %	9.9	24.2	
Adjusted OR (95% CI)*	Ref	2.32 (1.9–2.84)	
AKI-D			
Incidence, %	0.3	0.6	
Adjusted OR (95% CI)*	Ref	1.6 (0.58–4.42)	0.37
Shock state			
Incidence, %	1.9	7.8	
Adjusted OR (95% CI)*	Ref	3.21 (2.35–4.39)	
Bleeding			
Incidence, %	6.4	16.9	
Adjusted OR (95% CI)*	Ref	2.55 (2.05–3.17)	
DIC			
Incidence, %	0.1	0.2	
Adjusted OR (95% CI)*	Ref	0.8 (0.11–6.01)	0.83
Acute respiratory failure			
Incidence, %	7.4	18	
Adjusted OR (95% CI)*	Ref	2.22 (1.8–2.76)	
Stroke			
Incidence, %	0.4	0.5	
Adjusted OR (95% CI)*	Ref	0.76 (0.24–2.39)	0.64
Mechanical ventilation for >96 hours			
Incidence, %	1.2	6	
Adjusted OR (95% CI)*	Ref	4.33 (3.05–6.16)	
Length of stay			
Mean ±SE	5.4 ±0.03	13.7 ±0.6	
Adjusted parameter estimate (95% CI)*†	Ref	2.10 (1.98–2.24)	
Average hospital costs			
Mean ±SE	55,193 ±764	147,356.5 ±12,734.1	
Adjusted parameter estimate (95% CI)*†	Ref	2.01 (1.87–2.17)	

**Figure 1:**
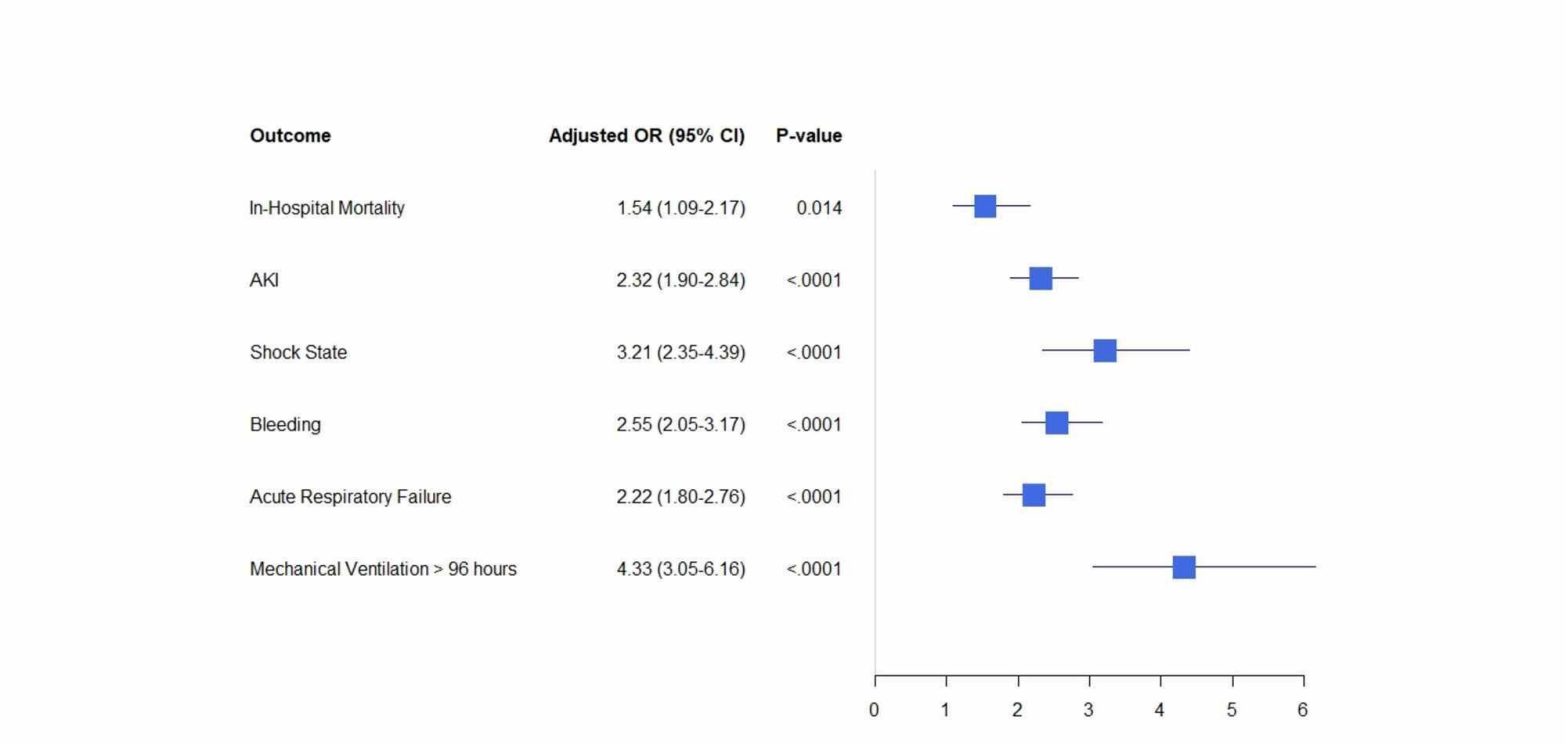
Adjusted odds ratio for in-hospital outcomes in VTE hospitalizations with Clostridium difficile infections VTE: venous thromboembolism; OR: odds ratio; CI: confidence interval; AKI: acute kidney injury

Notably, CDI in VTE hospitalizations was independently associated with increased risk of bleeding, acute kidney injury (AKI), shock state, acute respiratory failure requiring mechanical ventilation for >96 hours (p: <0.001 for all; adjusted for age, race, sex, insurance status, hospital characteristics, and all significant comorbidities listed in Table 1) (Figure 1).

Subgroup analyses of DVT vs. PE hospitalizations were also performed. For both groups, hospitalizations with concomitant CDI had an increased risk of AKI, bleeding, and acute respiratory failure needing mechanical ventilation for >96 hours (p: <0.001 for all, adjusted for age, race, sex, insurance status, hospital characteristics, and all significant comorbidities listed in Table 1) (Table 3).

**Table 3:** In-hospital outcomes in DVT/PE hospitalizations with and without Clostridium difficile infections *Adjusted for age, race, sex, insurance status, hospital characteristics, and all significant comorbidities listed in Table 1 †Parameter estimates represent the antilog of the b regression coefficients obtained from the log-transformed regression models PE: pulmonary embolism; DVT: deep vein thrombosis; OR: odds ratio; CI: confidence interval; AKI: acute kidney injury; AKI-D: acute kidney injury requiring dialysis; DIC: disseminated intravascular coagulation; SE: standard error

Variables	PE hospitalizations			DVT hospitalizations		
	No Clostridium difficile infections	Clostridium difficile infections	P-value	No Clostridium difficile infections	Clostridium difficile infections	P-value
In-hospital mortality						
Incidence, %	4.5	7.9		0.8	1.9	
Adjusted OR (95% CI)*	Ref	1.39 (0.97–2.0)	0.072	Ref	1.85 (0.57–5.96)	0.31
AKI						
Incidence, %	11.7	24.7		7	23.7	
Adjusted OR (95% CI)*	Ref	2.07 (1.63–2.62)		Ref	2.97 (1.97–4.48)	
AKI-D						
Incidence, %	0.4	0.2		0.2	1.9	
Adjusted OR (95% CI)*	Ref	0.48 (0.07–3.5)	0.47	Ref	6.38 (1.83–22.15)	0.0036
Shock state						
Incidence, %	3	10.6		0.3	1.3	
Adjusted OR (95% CI)*	Ref	2.97 (2.15–4.11)		Ref	2.51 (0.59–10.62)	0.2122
Bleeding						
Incidence, %	7.4	18.7		5.3	14.1	
Adjusted OR (95% CI)*	Ref	2.47 (1.92–3.17)		Ref	2.69 (1.7–4.27)	
DIC						
Incidence, %	0.2	0.2		0.0004	0	
Adjusted OR (95% CI)*	Ref	0.91 (0.12–6.95)	0.93	Ref	NA	NA
Acute respiratory failure						
Incidence, %	11.2	23.3		1.3	5.1	
Adjusted OR (95% CI)*	Ref	1.99 (1.58–2.51)		Ref	2.73 (1.24–5.99)	0.0125
Stroke						
Incidence, %	0.6	0.2		0.1	1.3	
Adjusted OR (95% CI)*	Ref	0.24 (0.03–1.75)	0.16	Ref	6.21 (1.34–28.73)	0.0195
Mechanical ventilation for >96 hours						
Incidence, %	1.8	8.1		0.2	1.3	
Adjusted OR (95% CI)*	Ref	3.93 (2.72–5.67)		Ref	4.5 (1.02–19.8)	0.0464
Length of stay						
Mean ±SE	5.9 ±0.05	14.5 ±0.8		5.0 ±0.04	12.5 ±1.2	
Adjusted parameter estimate (95% CI)*†	Ref	2.05 (1.88–2.23)		Ref	1.99 (1.76–2.24)	
Average hospital costs						
Mean ±SE	60,903.1 ±985.7	166,302.7 ±17,358.8		47,306.4 ±655	108,513.9 ±12,478	
Adjusted parameter estimate (95% CI)*†	Ref	2.16 (2.00–2.33)		Ref	1.84 (1.59–2.14)	

**Figure 2:**
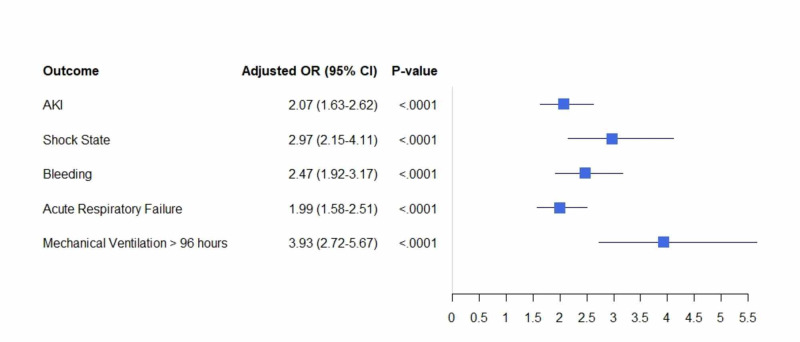
Adjusted odds ratio for in-hospital outcomes in PE hospitalizations with Clostridium difficile infections PE: pulmonary embolism; AKI: acute kidney injury; OR: odds ratio; CI: confidence interval

**Figure 3:**
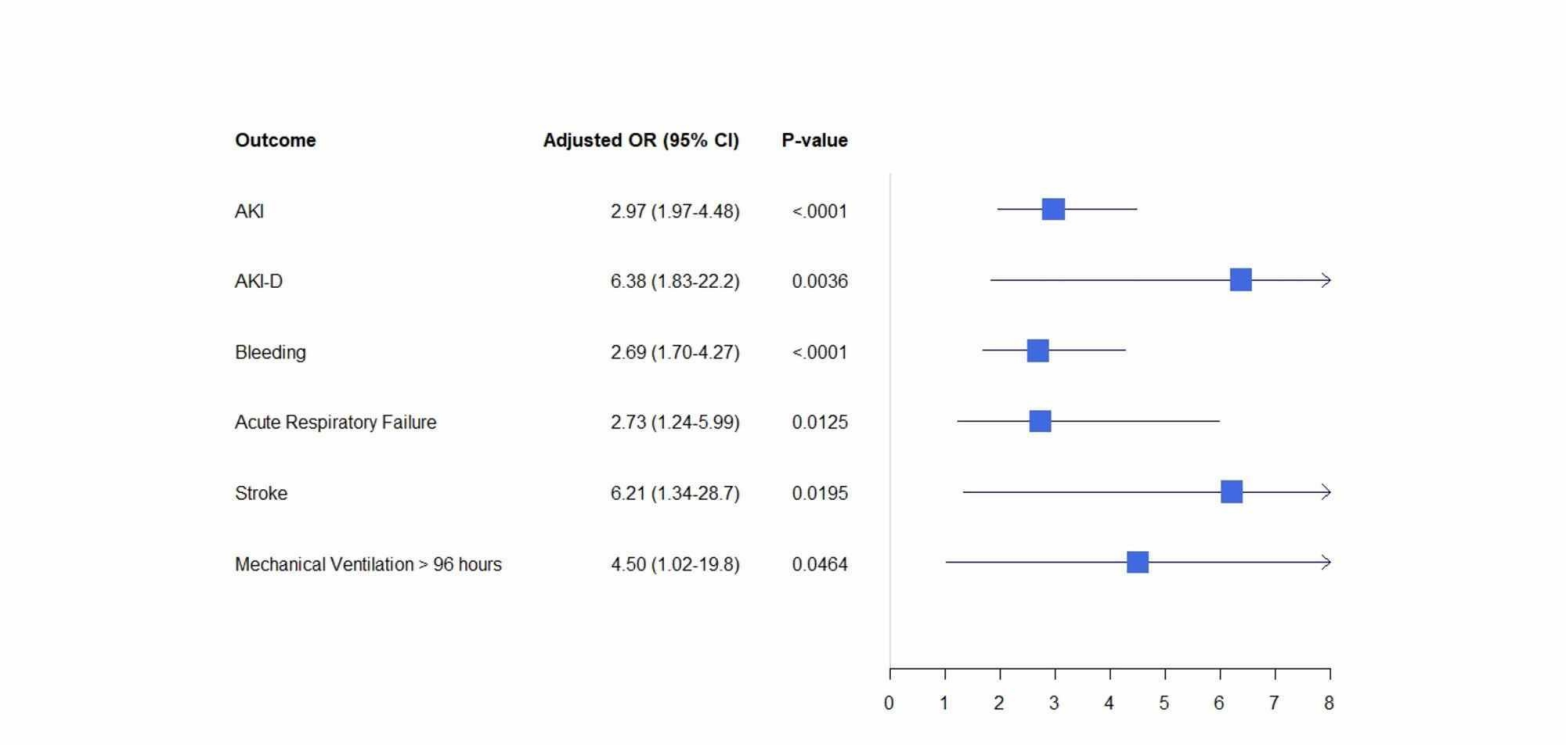
Adjusted odds ratio for in-hospital outcomes in DVT hospitalizations with Clostridium difficile infections DVT: deep vein thrombosis; AKI: acute kidney injury; AKI-D: acute kidney injury requiring dialysis; OR: odds ratio; CI: confidence interval

The odds of shock were greater in CDI and PE group (10.6% vs. 3%, adjusted OR: 2.97, 95% CI: 2.15-4.11, p: <0.001) (Figure 2), whereas the odds of stroke were higher in hospitalizations with CDI and DVT (1.3% vs. 0.1%, adjusted OR: 6.21, 95% CI: 1.5-25.71, p: <0.01) (Table 3) (Figure 3).

## Discussion

CDIs pose a significant burden on healthcare systems, not just in the United States but across the world [6]. Past studies have demonstrated a two-fold increase in the incidence of CDI among hospitalized adults in the United States between 2001-2010 [2]. Data from 2012 showed that annual cost for management of CDI amounted to approximately $800 million in the United States and €3,000 million in Europe, barring the costs for secondary complications [7]. While the rate of healthcare-associated CDI decreased in the United States between 2011-2017 by an estimated 36%, the rate of community-associated CDI has not changed in that time and now accounts for nearly half of all infections [8]. CDI has also been shown to have an independent association with the development of VTE, and findings of associations between acute infections and increased risk of VTE support the hypothesis that CDI may be causing a proinflammatory, procoagulant state in the human body [9-11].

While the association between CDI and increased risk of VTE is known, data regarding the impact of CDI on outcomes of these cases remain scarce. Our study, to the best of our knowledge, is the first of its kind to assess the outcomes of VTE in the presence of CDI, and the data we present here suggest that the presence of CDI independently causes a substantial increase in healthcare costs. This can be seen directly in terms of increased raw cost of VTE hospitalizations, and also in terms of increased mean length of stay, AKI, bleeding complications, shock, and the need for mechanical ventilation.

Fully understanding the burden CDI places on healthcare is critical to ensure adequate allocation of resources to CDI treatment and prevention efforts. Also, understanding its impact in the setting of DVT and PE admissions, themselves sources of considerable morbidity and healthcare cost, would seem essential. This is of particular importance when considering the apparent success of infection control programs and reduced prescriptions of fluoroquinolones in reducing the rates of healthcare-associated CDI, suggesting that there are interventions whose widespread adoption could further reduce rates of CDI and its associated complications [12].

Many of the clinical outcomes presented here can be attributed to CDI, causing a systemic proinflammatory state, with hypovolemia secondary to gastrointestinal losses, and third spacing secondary to inflammation. This drives outcomes like shock, AKI, and the need for dialysis. CDI patients with VTE outcomes seem to have more underlying comorbidities in general, and these, in turn, may put them at a higher risk of prolonged hospital stay and poor clinical outcomes. However, the adverse outcomes mentioned here were found after adjusting for these comorbidities, suggesting that CDI is an independent cause of serious adverse events in VTE hospitalizations.

Both the DVT and PE subgroups showed an increased risk of the need for prolonged mechanical ventilation (>96 hours) for CDI hospitalizations, which is somewhat surprising. In PE patients, it might reflect a combination of both ventilation/perfusion (V/Q) mismatch from PE and systemic inflammatory state from CDI. However, the presence of the effect in DVT hospitalizations, with a comparable OR (3.93 in PE patients, 4.5 in DVT patients), suggests that the presence of thrombotic events marks greater disease severity in CDI. Though speculative, it prompts a need for further investigation, both in terms of research, and a higher index of suspicion for thrombotic events in CDI hospitalizations. The elevated risk of shock in hospitalizations with PE and CDI versus PE without CDI is less surprising and might be explained by the addition of obstructive elements to existing hypovolemic and distributive shock from CDI. Interestingly, the population with VTE but no CDI had a higher prevalence of hyperlipidemia, obesity, smoking, and prior VTE. All of these are independent risk factors for VTE, and their decreased prevalence in the VTE with CDI group further supports the theory that CDI is a separate cause of thrombotic disease burden. Further studies are warranted to determine the causality of these outcomes, which may impact how we approach treating patients with CDI while being cognizant of the possible complications caused by VTEs in this high-risk population. As for the marked increase in the incidence of stroke noted among CDI hospitalizations with DVT, which was absent among hospitalizations with CDI and PE, it is challenging to explain. While risk factors for arterial and venous thrombotic diseases are shared, the stroke itself being a notorious risk factor for the development of recurrent CDIs, and for VTE, the risks of developing PE and DVT after stroke are very similar [13]. This may be a fruitful area for further study.

Our study has a few limitations. Firstly, our analysis was retrospective in nature. Hence, it was difficult to ascertain the causal relationship between CDI and VTE. Secondly, given the observational nature of the study, we found it hard to identify and adjust for all possible confounders. Thirdly, as a database study, it was implausible to determine with certainty if a specific diagnosis had been made during the hospitalization of record or if a patient had carried a history of such a diagnosis. Lastly, one NIS entry is equivalent to one hospitalization. Hence, a single patient may account for multiple entries if hospitalized more than once within the study period. However, despite these limitations, we believe the results still highlight the significance of CDI infections in patients admitted for VTE.

## Conclusions

Our study, to the best of our knowledge, is the first study of its kind to assess the outcomes of VTE in the presence of CDI. CDI remains a considerable burden in terms of increased length of hospital stay and healthcare costs, and it independently increases the risk of in-hospital mortality and various complications in VTE patients. Taking adequate steps to prevent CDI in the VTE population may avert unforeseen complications, improve in-hospital outcomes, and reduce healthcare costs.

## Appendices

**Table 4:** ICD 10 codes ICD 10: International Classification of Diseases 10th Revision; DIC: disseminated intravascular coagulation

Deep vein thrombosis	Pulmonary embolism	Clostridium difficile infections	Smoking	Alcohol abuse	Prior venous thromboembolism	Hyperlipidemia	Obesity	Congestive heart failure	Peripheral vascular disease	Diabetes, uncomplicated	DIC
I82401'	I2692'	A047'	F17200'	F1010'	Z86718'	E785'	'E6601'	I099	I700'	E100	D65'
I82402'	I2602'	A0471'	F17201'	F1011'	I82701'		'E6609'	I110	I701'	E101	
I82403'	I2609'	A0472'	F17203'	F10120'	I82702'		'E661'	I130	I70201'	E109	
I82409'	I2699'		F17208'	F10121'	I82703'		'E662'	I132	I70202'	E110	
I82411'			F17209'	F10129'	I82709'		'E663'	I255	I70203'	E111	
I82412'			F17210'	F1014'	I82711'		'E668'	I420	I70208'	E119	
I82413'			F17211'	F10150'	I82712'		'E669'	I425	I70209'	E120	
I82419'			F17213'	F10151'	I82713'		'Z6825'	I429	I70211'	E121	
I82421'			F17218'	F10159'	I82719'		'Z6826'	I43	I70212'	E129	
I82422'			F17219'	F10180'	I82721'		'Z6827'	P290	I70213'	E130	
I82423'			F17220'	F10181'	I82722'		'Z6828'	I501'	I70218'	E131	
I82429'			F17221'	F10182'	I82723'		'Z6829'	I5020'	I70219'	E139	
I82431'			F17223'	F10188'	I82729'		'Z6830'	I5021'	I70221'	E140	
I82432'			F17228'	F1019'	I82501'		'Z6831'	I5022'	I70222'	E141	
I82433'			F17229'		I82502'		'Z6832'	I5023'	I70223'	E149	
I82439'			F17290'		I82503'		'Z6833'	I5030'	I70228'	E1100'	
I82441'			F17291'		I82509'		'Z6834'	I5031'	I70229'	E1101'	
I82442'			F17293'		I82511'		'Z6835'	I5032'	I70231'		
I82443'			F17298'		I82512'		'Z6836'	I5033'	I70232'		
I82449'			F17299'		I82513'		'Z6837'	I5040'	I70233'		
I82491'					I82519'		'Z6838'	I5041'	I70234'		
I82492'					I82521'		'Z6839'	I5042'	I70235'		
I82493'					I82522'		'Z6841'	I5043'	I70238'		
I82499'					I82523'		'Z6842'	I50810'	I70239'		
I824Y1'					I82529'		'Z6843'	I50811'	I70241'		
I824Y2'					I82531'		'Z6844'	I50812'	I70242'		
I824Y3'					I82532'		'Z6845'	I50813'	I70243'		
I824Y9'					I82533'			I50814'	I70244'		
I824Z1'					I82539'			I5082'	I70245'		
I824Z2'					I82541'			I5083'	I70248'		
I824Z3'					I82542'			I5084'	I70249'		
I824Z9'					I82543'			I5089'	I7025'		
I82A11'					I82549'			I509'	I70291'		
I82601'					I82591'				I70292'		
I82602'					I82592'				I70293'		
I82603'					I82593'				I70298'		
I82609'					I82599'				I70299'		
I82611'					I825Y1'				I708'		
I82612'					I825Y2'				I7090'		
I82613'					I825Y3'				I7091'		
I82619'					I825Y9'				I7092'		
I82621'					I825Z1'				I7100'		
I82622'					I825Z2'				I7101'		
I82623'					I825Z3'				I7102'		
I82629'					I825Z9'				I7103'		
I82A12'					I82701'				I711'		
I82A13'					I82702'				I712'		
I82A19'					I82703'				I713'		
I82C11'					I82709'				I714'		
I82C12'					I82711'				I715'		
I82C13'					I82712'				I716'		
I82B11'					I82713'				I718'		
I82B12'					I82719'				I719'		
I82B13'					I82721'				I731		
I82B19'					I82722'				I738		
I82C19'					I82723'				I739		
					I82729'				I771		
					I82891'				I790		
					I82A21'				I792		
					I82A22'				K551		
					I82A23'				K558		
					I82A29'				K559		
					I82B21'				Z958		
					I82B22'				Z959		
					I82B23'						
					I82B29'						

**Table 5:** ICD 10 codes continued ICD 10: International Classification of Diseases 10th Revision

Diabetes, complicated	Hypertension	Chronic kidney disease		Acute kidney injury		Surgery		Sepsis	Shock state		Acute kidney injury + dialysis		Bleeding		Paralysis	Acute respiratory failure			Stroke	
E1021'	'I10'	N181		N178		Y830'		A4101'	R570'		N186'		D62'		G8100'	J9601'			I6300'	
E1022'	'I110'	N182		N170		Y831'		A4102'	R571'		Z992'		I6000'		G8101'	J9602'			I63011'	
E1029'	'I119'	N183		N171		Y832'		A411'	R578'		N178		I6001'		G8102'	J9600'			I63012'	
E10311'	'I120'	N184		N172		Y833'		A412'	R579'		N170		I6002'		G8103'	J9620'			I63013'	
E10319'	'I129'	N186		N179		Y834'		A413'	R6521'		N171		I6010'		G8104'	J9621'			I63019'	
E10321'	'I130'	N185				Y835'		A414'	T8110XA'		N172		I6011'		G8110'	J9622'			I6302'	
E103211'	'I1310'	N189				Y836'		A4150'	T8110XD'		N179		I6012'		G8111'	J9690'			I63031'	
E103212'	'I1311'	I1310'				Y838'		A4151'	T8110XS'				I602'		G8112'	J9691'			I63032'	
E103213'	'I132'					Y839'		A4152'	T8111XA'				I6020'		G8113'	J9692'			I63033'	
E103219'	'I150'							A4153'	T8111XD'				I6021'		G8114'	R0603			I63039'	
E10329'	'I151'							A4159'	T8111XS'				I6022'		G8190'	J80			I6309'	
E103291'	'I152'							A4181'	T8112XA'				I6030'		G8191'	J810			I6310'	
E103292'	'I158'							A4189'	T8112XD'				I6031'		G8192'	J95821			I63111'	
E103293'	'I159'							A419'	T8112XS'				I6032'		G8193'	R092			I63112'	
E103299'								R6520'	T8119XA'				I604'		G8194'	R0609			I63113'	
E10331'									T8119XD'				I6050'		G8220'	R0689			I63119'	
E103311'									T8119XS'				I6051'		G8221'				I6312'	
E103312'													I6052'		G8222'				I63131'	
E103313'													I606'		G8250'				I63132'	
E103319'													I607'		G8251'				I63133'	
E10339'													I608'		G8252'				I63139'	
E103391'													I609'		G8253'				I6319'	
E103392'													I610'		G8254'				I6320'	
E103393'													I611'		G830'				I63211'	
E103399'													I612'		G8310'				I63212'	
E10341'													I613'		G8311'				I63213'	
E103411'													I614'		G8312'				I63219'	
E103412'													I615'		G8313'				I6322'	
E103413'													I616'		G8314'				I63231'	
E103419'													I618'		G8320'				I63232'	
E10349'													I619'		G8321'				I63233'	
E103491'													I6200'		G8322'				I63239'	
E103492'													I6201'		G8323'				I6329'	
E103493'													I6202'		G8324'				I6330'	
E103499'													I6203'		G8330'				I63311'	
E10351'													I621'		G8331'				I63312'	
E103511'													I629'		G8332'				I63313'	
E103512'													H35731'		G8333'				I63319'	
E103513'													H35732'		G8334'				I63321'	
E103519'													H35733'		G834'				I63322'	
E103521'													H35739'		G801'				I63323'	
E103522'													H3560'		G802'				I63329'	
E103523'													H3561'		G041'				I63331'	
E103529'													H3562'		G800'				I63332'	
E103531'													H3563'		G114'				I63333'	
E103532'													H31301'						I63339'	
E103533'													H31302'						I63341'	
E103539'													H31303'						I63342'	
E103541'													H31309'						I63343'	
E103542'													H31311'						I63349'	
E103543'													H31312'						I6339'	
E103549'													H31313'						I6340'	
E103551'													H31319'						I63411'	
E103552'													H31411'						I63412'	
E103553'													H31412'						I63413'	
E103559'													H31413'						I63419'	
E10359'													H31419'						I63421'	
E103591'													H47021'						I63422'	
E103592'													H47022'						I63423'	
E103593'													H47023'						I63429'	
E103599'													H47029'						I63431'	
E1036'													H4310'						I63432'	
E1037X1'													H4311'						I63433'	
E1037X2'													H4312'						I63439'	
E1037X3'													H4313'						I63441'	
E1037X9'													I621'						I63442'	
E1039'													I312						I63443'	
E1040'													S40011A'						I63449'	
E1041'													S40011D'						I6349'	
E1042'													S40011S'						I6350'	
E1043'													S40012A'						I63511'	
E1044'													S40012D'						I63512'	
E1049'													S40012S'						I63513'	
E1051'													S40019A'						I63519'	
E1052'													S40019D'						I63521'	
E1059'													S40019S'						I63522'	
E10610'													S40021A'						I63523'	
E10618'													S40021D'						I63529'	
E10620'													S40021S'						I63531'	
E10621'													S40022A'						I63532'	
E10622'													S40022D'						I63533'	
E10628'													S40022S'						I63539'	
E10630'													S40029A'						I63541'	
E10638'													S40029D'						I63542'	
E10641'													S40029S'						I63543'	
E10649'													M79A11'						I63549'	
E1065'													M79A12'						I6359'	
E1069'													M79A19'						I636'	
E108'													M79A21'						I638'	
E1121'													M79A22'						I6381'	
E1122'													M79A29'						I6389'	
E1129'													M79A3'						I639'	
E11311'													M79A9'						I6601'	
E11319'																			I6602'	
E11321'																			I6603'	
E113211'																			I6609'	
E113212'																			I6611'	
E113213'																			I6612'	
E113219'																			I6613'	
E11329'																			I6619'	
E113291'																			I6621'	
E113292'																			I6622'	
E113293'																			I6623'	
E113299'																			I6629'	
E11331'																			I663'	
E113311'																			I668'	
E113312'																			I669'	
E113313'																				
E113319'																				
E11339'																				
E113391'																				
E113392'																				
E113393'																				
E113399'																				
E11341'																				
E113411'																				
E113412'																				
E113413'																				
E113419'																				
E11349'																				
E113491'																				
E113492'																				
E113493'																				
E113499'																				
E11351'																				
E113511'																				
E113512'																				
E113513'																				
E113519'																				
E113521'																				
E113522'																				
E113523'																				
E113529'																				
E113531'																				
E113532'																				
E113533'																				
E113539'																				
E113541'																				
E113542'																				
E113543'																				
E113549'																				
E113551'																				
E113552'																				
E113553'																				
E113559'																				
E11359'																				
E113591'																				
E113592'																				
E113593'																				
E113599'																				
E1136'																				
E1137X1'																				
E1137X2'																				
E1137X3'																				
E1137X9'																				
E1139'																				
E1140'																				
E1141'																				
E1142'																				
E1143'																				
E1144'																				
E1149'																				
E1151'																				
E1152'																				
E1159'																				
E11610'																				
E11618'																				
E11620'																				
E11621'																				
E11622'																				
E11628'																				
E11630'																				
E11638'																				
E11641'																				
E11649'																				
E1165'																				
E1169'																				
E118'																				
E1321'																				
E1322'																				
E1329'																				
E13311'																				
E13319'																				
E13321'																				
E133211'																				
E133212'																				
E133213'																				
E133219'																				
E13329'																				
E133291'																				
E133292'																				
E133293'																				
E133299'																				
E13331'																				
E133311'																				
E133312'																				
E133313'																				
E133319'																				
E13339'																				
E133391'																				
E133392'																				
E133393'																				
E133399'																				
E13341'																				
E133411'																				
E133412'																				
E133413'																				
E133419'																				
E13349'																				
E133491'																				
E133492'																				
E133493'																				
E133499'																				
E13351'																				
E133511'																				
E133512'																				
E133513'																				
E133519'																				
E133521'																				
E133522'																				
E133523'																				
E133529'																				
E133531'																				
E133532'																				
E133533'																				
E133539'																				
E133541'																				
E133542'																				
E133543'																				
E133549'																				
E133551'																				
E133552'																				
E133553'																				
E133559'																				
E13359'																				
E133591'																				
E133592'																				
E133593'																				
E133599'																				
E1336'																				
E1337X1'																				
E1337X2'																				
E1337X3'																				
E1337X9'																				
E1339'																				
E1340'																				
E1341'																				
E1342'																				
E1343'																				
E1344'																				
E1349'																				
E1351'																				
E1352'																				
E1359'																				
E13610'																				
E13618'																				
E13620'																				
E13621'																				
E13622'																				
E13628'																				
E13630'																				
E13638'																				
E13641'																				
E13649'																				
E1365'																				
E1369'																				
E138'																				
