# Correction: The Impact of Clostridium Difficile Infections on In-Hospital Outcomes of Venous Thromboembolism (Deep Vein Thrombosis or Pulmonary Embolism) Hospitalizations

**DOI:** 10.7759/cureus.c35

**Published:** 2020-09-01

**Authors:** Khushali Jhaveri, Aniruddh Som, Sandeep A Padala, Salim Surani

**Affiliations:** 1 Internal Medicine, The Georgetown University Hospital/MedStar Washington Hospital Center, Washington, DC, USA; 2 Internal Medicine, Medstar Washington Hospital Center-Georgetown University Hospital, Washington, DC, USA; 3 Nephrology, Medical College of Georgia - Augusta University, Augusta, USA; 4 Internal Medicine, Corpus Christi Medical Center, Corpus Christi, USA; 5 Internal Medicine, University of North Texas, Dallas, USA

Due to a technical error with the Cureus software, all instances of P-values measuring "< .0001" were erroneously omitted from Tables 1-3. These p-values have been restored to the tables. Cureus sincerely regrets the technical and editorial errors that led to this article being published without the p-values.

